# Managerial leadership and ischaemic heart disease among employees: the Swedish WOLF study

**DOI:** 10.1136/oem.2008.039362

**Published:** 2008-12-11

**Authors:** A Nyberg, L Alfredsson, T Theorell, H Westerlund, J Vahtera, M Kivimäki

**Affiliations:** 1 Department of Public Health Sciences, Karolinska Institute, SE–171 77 Stockholm, Sweden; 2 Stress Research Institute, Stockholm University, Stockholm, Sweden; 3 Institute of Environmental Medicine, Karolinska Institute, Stockholm, Sweden; 4 Stockholm Center of Public Health, Karolinska University Hospital, Stockholm, Sweden; 5 Finnish Institute of Occupational Health, Helsinki, Finland; 6 Department of Epidemiology and Public Health, University College London, London, UK

## Abstract

**Objective:**

To investigate the association between managerial leadership and ischaemic heart disease (IHD) among employees.

**Methods:**

Data on 3122 Swedish male employees were drawn from a prospective cohort study (WOLF). Baseline screening was carried out in 1992–1995. Managerial leadership behaviours (consideration for individual employees, provision of clarity in goals and role expectations, supplying information and feedback, ability to carry out changes at work successfully, and promotion of employee participation and control) were rated by subordinates. Records of employee hospital admissions with a diagnosis of acute myocardial infarction or unstable angina and deaths from IHD or cardiac arrest to the end of 2003 were used to ascertain IHD. Cox proportional-hazards analyses were used to calculate hazard ratios for incident IHD per 1 standard deviation increase in standardised leadership score.

**Results:**

74 incident IHD events occurred during the mean follow-up period of 9.7 years. Higher leadership score was associated with lower IHD risk. The inverse association was stronger the longer the participant had worked in the same workplace (age-adjusted hazard ratio 0.76 (95% CI 0.61 to 0.96) for employment for 1 year, 0.77 (0.61 to 0.97) for 2 years, 0.69 (0.54 to 0.88) for 3 years, and 0.61 (0.47 to 0.80) for 4 years); this association was robust to adjustments for education, social class, income, supervisory status, perceived physical load at work, smoking, physical exercise, BMI, blood pressure, lipids, fibrinogen and diabetes. The dose–response association between perceived leadership behaviours and IHD was also evident in subsidiary analyses with only acute myocardial infarction and cardiac death as the outcome.

**Conclusion:**

If the observed associations were causal then workplace interventions should focus on concrete managerial behaviours in order to prevent IHD in employees.

A recent meta-analysis of prospective cohort studies examining determinants of cardiovascular disease, suggests an average excess risk of 50% in employees who are exposed to an adverse psychosocial work environment (eg, high job strain).^1^ These results have considerable clinical implications, especially since psychosocial stressors at work are relatively common.^2^

Before a consensus on psychosocial work environment as a major risk factor for cardiovascular disease can be reached, data from population interventions aimed at preventing this risk factor are needed. To date, no large scale evaluations of such interventions have been published, perhaps due to lack of knowledge regarding how such interventions should be carried out. To help define the interventions required, we tested whether concrete managerial behaviours promoting a favourable psychosocial work environment were associated with a reduced risk of cardiovascular disease among employees.

Observational evidence on various general conceptualisations describing leadership as predictors of employee health is accumulating.^3^ For example, considerate behaviour on behalf of the leader, structures initiated with consideration for the employee, and transformational behaviours (communication of a vision, intellectual stimulation, consideration of individual employees) have all been found to be related to good employee health, job satisfaction and productivity.^4^^–^13 A high level of justice in managerial behaviours has been shown to be related to increased employee motivation and cooperation, decreased levels of negative emotions and sickness absence, and reduced risk of coronary heart disease (CHD).^14^^–^20 In contrast, perceived abusive, passive-avoidant and laissez-faire leadership has been found to be associated with increased psychological distress,^21^^–^23 and lack of social support in combination with job strain has been shown to be related to elevated risks for cardiovascular disease and increased levels of sickness absence.^24^^–^26 Finally, a Swedish study found a decrease in serum cortisol levels and an improvement in perceived authority over decisions among employees whose managers participated in a psychosocial manager program compared with a control group.^27^

Many of the studies described above were based on cross-sectional designs, self-reported data and/or assessments of managerial leadership defined in general terms of justice and support. The aim of the present study was to examine the association between employees’ perceptions of managerial behaviours and objectively measured incident ischaemic heart disease (IHD) in a prospective research design, while adjusting for conventional cardiovascular risk factors. We focussed on concrete managerial behaviours, such as the manager’s consideration for the individual employee, provision of clarity in goals and role expectations, supplying information and feedback, ability to carry out changes at work successfully, and promotion of employee participation and control (for a description of the measured leadership behaviours, see table 4).

**Table 4:** Association of standardised leadership scale items with incident IHD among employees with a minimum 4-year exposure

Items		Participants, n (events, n)	Age-adjusted hazard ratio for IHD per 1 SD increase in leadership score (95% CI)
1. My boss gives me the information I need		1463 (44)	0.65 (0.50 to 0.83)
2. My boss is good at pushing through and carrying out changes		1441 (44)	0.61 (0.45 to 0.81)
3. My boss explains goals and subgoals for our work so that I understand what they mean for my particular part of the task		1453 (44)	0.61 (0.46 to 0.79)
4. I have a clear picture of what my boss expects of me		1454 (44)	0.77 (0.59 to 1.01)
5. My boss shows that he/she cares how things are for me and how I feel		1455 (44)	0.71 (0.54 to 0.93)
6. I have sufficient power in relation to my responsibilities		1442 (44)	0.64 (0.48 to 0.84)
7. My boss takes the time to become involved in his/her employees’ professional development		1447 (44)	0.69 (0.51 to 0.92)
8. My boss encourages my participation in the scheduling of my work		1446 (44)	0.84 (0.63 to 1.12)
9. I am praised by my boss if I have done something good		1452 (44)	0.55 to 0.97)
10. I am criticised by my boss if I have done something that is not good		1452 (44)	1.03 (0.77 to 1.38)

Response format: 1: “No, never”; 2: “No, seldom”; 3: “Yes, sometimes”; 4: “Yes, often”. Internal consistency (Cronbach α) = 0.86. IHD, ischaemic heart disease.

## METHODS

### Study population

Data were drawn from the WOLF (Work, Lipids, and Fibrinogen) Stockholm study, which is a prospective cohort study of employees aged 19–70 working in companies in the Stockholm area. Twenty occupational health units carried out the baseline screening between November 1992 and June 1995. Overall, 3239 men and 2459 women (76% participation) took part in a clinical examination and a questionnaire survey. Records of hospital admissions and deaths to the end of 2003 were obtained from national registers and were linked to the data. The Regional Research Ethics Board in Stockholm, and the ethics committee at Karolinska Institutet, Stockholm, Sweden approved the study (nos. 2006/158-31, 2008/240-32, 92-198 and 03-125).

We restricted the analyses to men only since there were too few cases of ischaemic disease among women (n = 12). Cases of prevalent ischaemic disease at baseline in 1992–1995 identified by hospital admission for ischaemic disease between 1963 and baseline screening were excluded from the analysis (21 men). An additional 46 men were excluded because they were above 65 years of age (official retirement age) at the start of the study, and finally 50 men were excluded because of missing data in the managerial leadership scale.

### Clinical characteristics

Education (low, intermediate, high) and smoking status (current smoker vs non-smoker) were self-reported, while income from work (in Swedish kronor) was obtained through registers. Systolic and diastolic blood pressure (mm Hg) was twice measured on the right arm in the supine position after 5 min rest with a 1 min interval. Height, weight and waist were measured to determine body mass index (BMI, kg/m^2^) and waist circumference (cm). Blood samples were taken after an overnight fast and analysed in the same laboratory (CALAB Medical Laboratories, Stockholm, Sweden) accredited by the Swedish Board for Accreditation and Conforming Assessment. Total cholesterol (mmol/l) and high density lipoprotein (HDL) cholesterol (mmol/l) were measured enzymatically after precipitation with phospotungstic acid and magnesium chloride. Low density lipoprotein (LDL) cholesterol concentration was calculated by the Friedewald formula. Fibrinogen in plasma (mmol/l) was determined by spectrometric test. The participants stated in the questionnaire whether they previously had had a heart attack, angina pectoris, chest pain at physical exertion or mental strain, heart failure, stroke, vascular spasms in calves (“window watcher syndrome”) or diabetes.

### Assessment of managerial leadership at work

The participants rated their managers’ behaviours using an assessment instrument which included 10 items with structured response scales (see table 4). These items constitute one dimension, leadership climate, of the psychosocial work environment measured in the Stress Profile. The Stress Profile is a validated instrument based upon consultation at work sites and established theories and research on work stress.^28^ The internal consistency for this scale was high (Cronbach α of 0.86) suggesting that a supervisor tends to either express all these behaviours or none of them. We summed the response scores and expressed this as a percentage of the theoretical maximum (100 refers to respondents with the highest score for every item of the scale; 0 refers to respondents with the lowest score for every item of the scale).

### Follow-up

Hard endpoint outcomes for IHD were defined as hospital admission with a main diagnosis registered as acute myocardial infarction (the International Classification of Diseases, version 9 (ICD-9) code 410; ICD-10 code I21) or unstable angina (ICD-9: 411; ICD-10: I20.0); or death with a registered underlying cause of IHD (ICD-9: 410–414; ICD-10: I20–I25) or cardiac arrest (ICD-9: 427; ICD-10: I46). Records of hospital admissions and deaths from 14 March 1963 until 31 December 2003 were obtained. Incident caseness was defined as the first event occurring after baseline screening, excluding prevalent cases at baseline.

### Statistical analysis

For each IHD outcome, the time to the event was defined as the number of days between baseline screening and the first diagnosis after baseline but before 31 December 2003. For employees with no events, the end of follow-up was 31 December 2003 or the date of death if earlier. Outcome of the primary analysis was a composite measure of acute myocardial infarction, unstable angina and cardiac death. Subsidiary analysis excluded unstable angina from the outcome to examine whether the association was seen with myocardial infarction and cardiac death only. We calculated age-adjusted hazard ratios with 95% confidence intervals from Cox proportional-hazards analyses for incident IHD per 1 standard deviation (SD) increase in standardised leadership score (mean 0, SD 1). Additional adjustments included socioeconomic characteristics and conventional risk factors. An interaction term between leadership and time worked in the current workplace was entered in a subsidiary analysis. SAS v 9.1 was used for the analyses.

## RESULTS

Descriptive statistics for the sample are shown in table 1. The participants were on average 42 years old and most of them were relatively highly educated and non-smokers. The comparatively favourable risk factor levels were due to the fact that all participants were employed, and that the sample was composed of employees with higher education than the average employee in Sweden and slightly better health care support than the average inhabitant in Stockholm.

**Table 1:** Sample characteristics: the WOLF Stockholm study

Risk factor at baseline	Men, n	Mean (SD) or %
Age, years	3122	41.6 (11.1)
Educational attainment		
High	1677	53.8
Intermediate	892	28.6
Low	547	17.6
Social class		
Professional and higher manager	632	23.3
Technical, lower management	727	26.8
Non-manual	239	8.8
Skilled manual	496	18.3
Unskilled manual	623	22.9
Supervisory status		
Yes	702	22.7
No	2386	77.3
Mean income per year, 1000	3104	253.11 (137.98)
Swedish kronor		
Perceived physical load at work (proxy quartiles)		
0–1	790	25.1
2–4	822	26.1
5–6	558	17.7
7–14	976	31.0
Physical exercise		
Never or very little	785	24.9
Now and then	1153	36.5
Regularly	1221	38.7
Smoking status		
Never smoker	1417	46.4
Ex-smoker	894	29.3
Current	742	24.3
Body mass index, kg/m^2^	3119	25.2 (3.3)
Systolic blood pressure, mm Hg	3118	123 (14)
Diastolic blood pressure, mm Hg	3118	75 (10)
Total cholesterol, mmol/l	3122	5.48 (1.15)
Total:HDL cholesterol ratio	3121	4.29 (1.45)
Triglycerides, mmol/l	3122	1.40 (1.02)
Fibrinogen, mmol/l	3121	2.69 (0.73)
Diabetes	3100	1.4

HDL, high density lipoprotein.

A total of 74 incident IHD events occurred during the mean follow-up time of 9.7 years (range: 3 days to 10.5 years). In age-adjusted analysis, a higher leadership score was associated with a lower IHD risk. This inverse association was dependent on the time worked in the current workplace (p = 0.049 for the interaction between leadership score and time worked in current workplace on incident IHD including unstable angina and p = 0.03 on acute myocardial infarction and cardiac death excluding unstable angina). As illustrated in table 2, the association was stronger the longer the participant had worked in the same workplace. This suggests a dose–response association between leadership and incident IHD.

**Table 2:** Dose–response association between managerial leadership and incident ischaemic heart disease among employees

Years at current workplace prior to survey (years)*	Men, n	Risk for incident IHD (including unstable angina)† per 1 SD increase in leadership score		Risk for incident IHD (excluding unstable angina)‡ per 1 SD increase in leadership score	
		Events, n	Hazard ratio (95% CI)	Events, n	Hazard ratio (95% CI)
Any amount of years	3122	74	0.80 (0.64 to 0.99)	54	0.79 (0.62 to 1.02)
At least 1 year	2423	63	0.76 (0.61 to 0.96)	44	0.72 (0.55 to 0.94)
At least 2 years	2012	58	0.77 (0.61 to 0.97)	40	0.73 (0.55 to 0.97)
At least 3 years	1768	51	0.69 (0.54 to 0.88)	34	0.66 (0.49 to 0.88)
At least 4 years	1468	44	0.61 (0.47 to 0.80)	29	0.55 (0.40 to 0.77)

Age-adjusted hazard ratio is per 1 SD increase in standardised leadership index score (mean 0, SD 1).

*Based on Statistic Sweden records (the number of years the participant had worked in the workplace prior to filling out the leadership questionnaire).

†Includes acute myocardial infarction (ICD-10: I21) or unstable angina (ICD-10: I20.0) necessitating hospitalisation or death from ischaemic heart disease (ICD-10:I20–I25) or cardiac arrest (ICD-10: I46).

‡Includes acute myocardial infarction (ICD-10: I21) necessitating hospitalisation or death from ischaemic heart disease (ICD-10: I20–I25) or cardiac arrest (ICD-10: I46).

IHD, ischaemic heart disease.

Table 3 presents the effects of multiple adjustments on the inverse association between leadership score and incident IHD among participants with complete data on all baseline characteristics and a minimum of 4 years in the current workplace. The association was robust to adjustments for socioeconomic factors and conventional risk factors for ischaemic disease. To assess possible reverse causality, we excluded those with self-reported angina, chest pain, vascular spasms in their calves, heart attack, heart failure or stroke at baseline (n = 172). In age-adjusted models of men with a minimum of 4 years in the current workplace, the hazard ratio for incident IHD was 0.63 (95% CI 0.46 to 0.86, p = 0.005).

**Table 3:** Association of standardised leadership score with incident IHD among employees after adjustment for different risk factors at baseline*

Adjustment variables in addition to age	Hazard ratio for IHD per 1 SD increase in leadership score(95% CI)
None	0.65 (0.49 to 0.87)
Education, supervisory status, social class, income	0.67 (0.49 to 0.90)
and physical load at work	
Smoking, physical exercise	0.65 (0.49 to 0.87)
BMI, systolic and diastolic blood pressure, total	0.61 (0.46 to 0.82)
cholesterol, total/HDL cholesterol ratio, triglycerides,	
fibrinogen, diabetes	
All of the above	0.63 (0.46 to 0.86)

*Only those with a minimum of 4-year exposure and no missing data in any of the predictors were included in these models (n = 1319, 40 events).

Ischaemic heart disease (IHD) included unstable angina. HDL, high density lipoprotein.

Finally, we examined the association of each leadership scale item with incident IHD (table 4). Except for three items (item 4: “I have a clear picture of what my boss expects of me”, item 8: “My boss encourages my participation in the scheduling of my work”, and item 10: “I am criticised by my boss if have done something that is not good”), all of the items in the leadership scale were significantly associated with incident IHD.

## DISCUSSION

This study of a contemporary cohort of working men in Sweden suggests an association between managerial leadership and incident IHD among employees, independent of a number of conventional risk factors. The managerial practices measured cover aspects of the manager’s consideration for the individual employee, provision of clarity in goals and role expectations, supplying information and feedback, ability to carry out changes at work successfully, and promotion of employee participation and control.

Our results are in agreement with previous studies on organisational justice and social support, that is, the extent to which people perceive that they are treated fairly and supported by their supervisors. Two independent occupational cohort studies showed a lower risk of incident CHD and cardiovascular mortality among employees who experienced a high level of organisational justice.^20^ ^29^ A small-scale study found that the ambulatory systolic blood pressure of employees with multiple supervisors was 15 mmHg lower on days worked under a supervisor perceived as fair than on days worked under an unfavourably perceived supervisor.^30^ A strength of the present study is the focus on very concrete managerial behaviours which may be useful when implementing workplace interventions.

In the leadership behaviours scale, the strongest predictors of IHD (with hazard ratios below 0.66) were items stating that the manager gives information and sufficient control to employees in relation to their responsibilities, explains goals and subgoals thoroughly, and is good at pushing through and carrying out changes. Employee preferences regarding managerial behaviours have been shown to differ between cultures, and the behaviours described above (ie, change-oriented and allowing a high degree of employee control) are in accordance with what is considered good managerial practice in Sweden.^31^^–^33 Highly educated employees were over-represented in this sample and more independence can be assumed to be preferred and successfully handled by such qualified personnel. It should not be forgotten that our findings were based on men only; the relationship may look different for women.

At least two items of the 10-item leadership behaviours scale are similar to the general conceptualisations of the Demand-Control Model (item 6) and social support (item 5). In our previous study of this same cohort, job strain (ie, high job demands combined with low job control) was associated with IHD, but not as strongly as managerial leadership was in the present study.^34^ The leadership scale shows a moderate correlation with the Demand-Control Questionnaire social support scale (Spearman’s correlation coefficient 0.44), suggesting that the leadership scale overlaps with the social support scale, but still has independent quality.^35^

Three items of the leadership behaviours scale (items 1, 5 and 9) were close to operationalisations of organisational justice and effort reward imbalance (validated measures of those concepts were unfortunately not available from the WOLF study). Regarding leadership theories, the leadership behaviours scale more strongly resembles task oriented and transactional leadership behaviours than relationship oriented and transformational leadership behaviours, although the latter is often seen as a stronger correlate of employees’ self-reported health.

Plausible mechanisms for the association between managerial leadership and CHD remain unclear. Recent publications on the relationship between managerial leadership and subordinate self-reported health have stressed the importance of negative aspects of managerial behaviours (eg, passive-avoidant and laissez-faire leadership).^22^ ^23^ Skogstad *et al* found laissez-faire leadership behaviours to be positively associated with employee role conflicts, role ambiguity and conflicts with co-workers. Psychosocial stress has been shown to increase the progression of coronary atherosclerosis.^36^ One could speculate that a present and active manager, providing structure, information and support, counteracts destructive processes in work groups, thereby promoting regenerative rather than stress-related physiological processes in employees.

We measured managerial behaviours using staff surveys; thus, the responses could reflect individual differences in perceiving leadership as well as actual behaviours exhibited by managers. A previous study has shown that work group perceptions of leadership differ from individual perceptions, and it was claimed that the latter better explain individuals’ health and sick leave.^37^ However, another study showed significant variance between work groups in perceptions of managerial leadership, and a strong average within-group agreement, indicating a homogeneity in perceptions among employees working under the same supervisor, not explained by individual characteristics.^38^ Individual perceptions could also to some extent reflect the fact that managers actually behave differently towards different employees since the relationship is a reciprocal process.^39^

**Main messages**

There is a prospective, dose–response relationship between concrete managerial behaviours and ischaemic heart disease among employees.

**Policy implications**

If the association between concrete managerial behaviours and ischaemic heart disease among employees is causal, then workplace health promotion interventions could focus on the managers’ concrete behaviours, such as the provision to employees of clear work objectives, information and sufficient power in relation to their responsibilities.

The number of years the respondents had been working for the same employer at the time of the survey was obtained from Statistics Sweden. This information provides an objective approximation of pre-survey exposure to the rated leadership qualities. The dose–response relationship between increased exposure time and decreased disease risk supports the possibility that the association between managerial behaviours and IHD reflects actual workplace influences rather than solely being determined by individual differences in perception. However, time worked for the same employer does not necessarily mean time worked for the same manager or supervisor. Changes in managers and supervisors may have occurred but are likely to dilute rather than inflate the observed effects.

Subjectivity bias in ascertainment of disease status is an increasingly recognised validity problem in studies on psychosocial factors and IHD. In a previous study of Scottish men, for example, the association between self-reported stress and CHD was largely driven by diagnoses based on symptom reporting.^40^ In the present study, the outcomes included only cardiovascular deaths and hospital admissions for diagnoses based on objective criteria (eg, ECG, enzymes, and CT or MRI imaging) of ischaemic disease. Conditions influenced by subjective reporting (eg, hospital admission from ill defined heart disease, haemorrhoids or “other” circulatory disorders) or self-reported data (eg, Rose angina) were excluded. Thus, bias due to misclassification of disease is an unlikely explanation of our findings.

In a study of Finnish middle-aged men, occupational, but not leisure time, physical activity was a strong predictor of 11-year progression of carotid atherosclerosis, especially in those with pre-existing IHD. Job strain had no significant effect after occupational physical activity was accounted for.^41^ Virkkunen *et al* found physical workload to be an important predictor of increased systolic blood pressure, which is a key pathway to CHD risk.^42^ Socioeconomic position is another potential confounder.^43^ In the present study, analyses were controlled for perceived physical load at work, physical exercise, and multiple indicators of socioeconomic position, including education, social class, income and supervisory status. We did not find evidence of major confounding, as the association between managerial leadership and employee IHD was little affected by these adjustments.

In conclusion, this is apparently the first study to provide evidence of a prospective, dose–response relationship between concrete managerial behaviours and objectively assessed IHD among employees. If the association is causal, this study suggests that interventions aimed at improving the psychosocial work environment and preventing ischaemic heart disease among employees could focus on concrete managerial behaviours, such as the provision of clear work objectives, information and sufficient control in relation to responsibilities.
